# Initial experience with vNOTES hysterectomy for benign conditions in a French university hospital

**DOI:** 10.52054/FVVO.14.2.018

**Published:** 2022-07-01

**Authors:** C Baron, A Netter, C Tourette, A Pivano, A Agostini, P Crochet

**Affiliations:** Department of Obstetrics and Gynecology, La Conception Hospital, Aix Marseille University, Marseille, France; Institut Méditerranéen de Biodiversité et d’Écologie marine et continentale (IMBE), Aix Marseille University, CNRS, IRD, Avignon University, Marseille, France; Department of Obstetrics and Gynecology, Charles Nicolle Hospital, University of Rouen, Rouen, France

**Keywords:** Hysterectomy, vNOTES, laparoscopic hysterectomy

## Abstract

**Background:**

Natural orifice transluminal endoscopic surgery by the vaginal route (vNOTES) is a new approach to performing hysterectomy. Clinical outcomes must be evaluated in centres that have started performing this technique.

**Objectives:**

To compare operative outcomes between vNOTES hysterectomy and laparoscopic hysterectomy during the introduction of the vNOTES approach in a teaching hospital.

**Material and Methods:**

A retrospective study was conducted from November 2019 to May 2021 at a French academic hospital in Marseille. The included patients underwent total hysterectomy for benign indications by vNOTES or conventional laparoscopy.

**Main outcome measures:**

Operative time, uterus weight, intraoperative complications, and postoperative complications according to the Clavien-Dindo classification.

**Results:**

Eighty-six patients underwent hysterectomy according to the selected criteria: 36 procedures were performed by vNOTES and 50 by laparoscopy. The mean operative time was shorter in the vNOTES group than in the laparoscopy group [116 min versus 149 min; p=0.003]. The mean uterus weight was not different between the vNOTES group and the laparoscopy group (238g versus 281g; p=0.572). Laparo-conversion occurred in one case in the vNOTES group (2.7%) and three cases in the laparoscopy group (3.4%). One Grade III postoperative complication occurred in the laparoscopy group, and no severe complication occurred in the vNOTES group.

**Conclusion:**

Operative outcomes of the vNOTES hysterectomy were favourable and support good feasibility without additional morbidity compared to laparoscopy.

**What is new?:**

During the introduction period of the vNOTES hysterectomy technique in a teaching hospital, reassuring operative outcomes and a low rate of complications were observed.

## Introduction

Hysterectomy is the most frequently performed procedure in gynaecological surgery worldwide, and nearly 90% of its indications are benign pathologies (Aarts et al., 2015). The four main approaches to this surgery are laparotomy, conventional laparoscopy, robot-assisted laparoscopy, and the vaginal route. The vaginal and laparoscopic approaches allow for reduced postoperative pain and length of hospital stay and a faster recovery compared to laparotomy. Additionally, the vaginal route has the advantage of producing no abdominal scars. The vaginal route is currently preferred in cases of benign pathologies (Aarts et al., 2015). However, the conventional vaginal route has disadvantages, including difficulty with exposure and visualisation of the operating field due to a narrow working space. Moreover, vaginal hysterectomy can be difficult in the cases of a large uterus that cannot be mobilised, even after caudo-cephalic dissection. Thus, the vaginal approach is currently being abandoned in France in favour of minimally invasive laparoscopic surgery (Chevrot et al., 2021).

Natural orifice transluminal endoscopic surgery (NOTES) technology was developed for trans gastric and transcolonic approaches. It was first investigated for the trans gastric approach in a porcine model (Kalloo et al., 2004). Vaginal NOTES (vNOTES) consist of a laparoscopy of the abdominopelvic cavity through the vaginal route. After the placement of a transvaginal device, the procedure is performed using conventional laparoscopic instruments. This technique was first explored in gynaecological surgery by Ahn et al. (2012) for benign adnexal conditions. The use of vNOTES has been explored in many other gynaecological procedures, including hysterectomy (Li and Hau, 2020; Housmans et al., 2020). Until now, vNOTES has been adopted by a small number of very experienced surgeons (Baekelandt et al., 2019). This technique is currently implemented by several gynaecological surgery teams around the world. The results obtained during this period of implementation must be evaluated.

The aim of the study was to compare the operative conditions and the postoperative follow-up observations for patients who underwent a hysterectomy for benign condition using vNOTES or using conventional laparoscopy during the introductory period of vNOTES in a university hospital.The aim of the study was to compare the operative conditions and the postoperative follow-up observations for patients who underwent a hysterectomy for benign condition using vNOTES or using conventional laparoscopy during the introductory period of vNOTES in a university hospital.

## Materials and Methods

This retrospective observational study was conducted during a 19-month period from November 2019 to May 2021 at the La Conception University Hospital in Marseille, France. Approval was obtained from the Institutional Review Board of the French College of Obstetricians and Gynecologists (CEROG 2021-GYN-0503). Women were eligible if they were aged at least 18 years, were undergoing a total hysterectomy with or without adnexectomy, and had been indicated for hysterectomy as a result of a benign condition. For these women, the surgeon decided that either of the following two approaches was possible: laparoscopy or vNOTES. The choice of the technique was based on patient preference. Patients were informed that vNOTES was a new technique and that they could opt instead for laparoscopy. The exclusion criteria for all patients were as follows: suspected endometriosis or oncologic condition, any suspicion of pelvic adhesions, and comorbidities contraindicating the application of pneumoperitoneum. During the study period, no patient meeting the inclusion criteria underwent surgery using the classical vaginal route according to the department’s policy to prioritise the implementation of vNOTES.

### Procedure

All procedures were performed during inpatient hospitalisation, in accordance with department policy.

Regarding vNOTES procedures, the woman was positioned in the lithotomy position. Antisepsis and draping included the perineum and the entire abdominal area to allow for emergency laparotomy or laparoscopic conversion, if necessary. Antibiotic prophylaxis of 2 g intravenous cefazolin was administered at the beginning of the procedure, and a Foley catheter was inserted.

**Figure 1:**
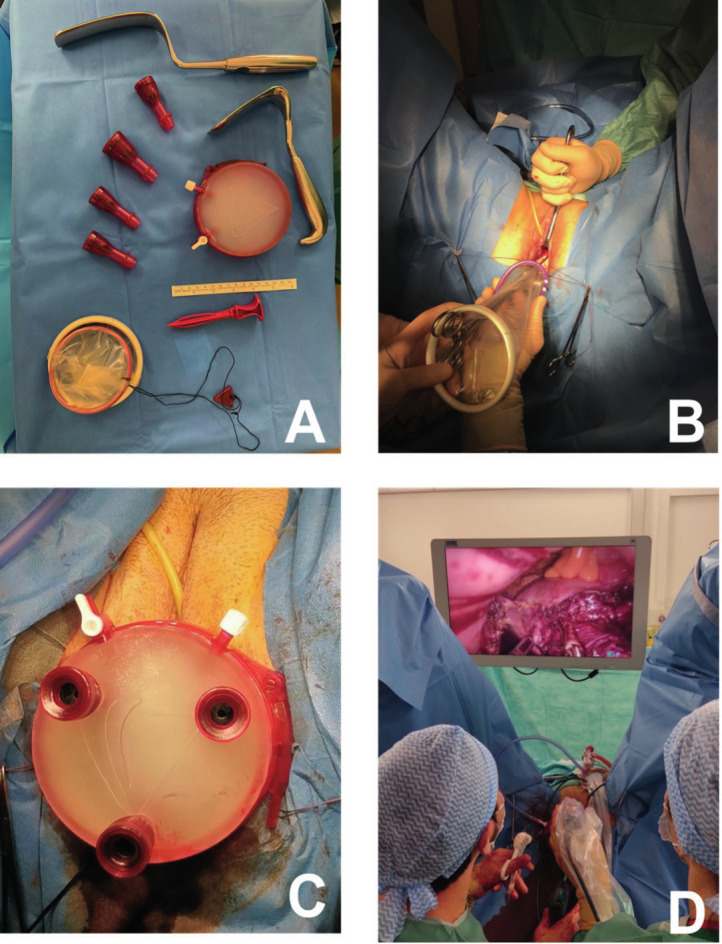
(A) GelPOINT™ V-Path transvaginal access platform 9.5cm and vaginal retractors for vNOTES hysterectomies. (B) Alexis’s retractor placement following the colpotomy step (C) Three trocars placed at 2, 6, and 10 o’clock on the GelPoint™ platform (D) Laparoscopic step of the vNOTES procedure.

**Figure 2:**
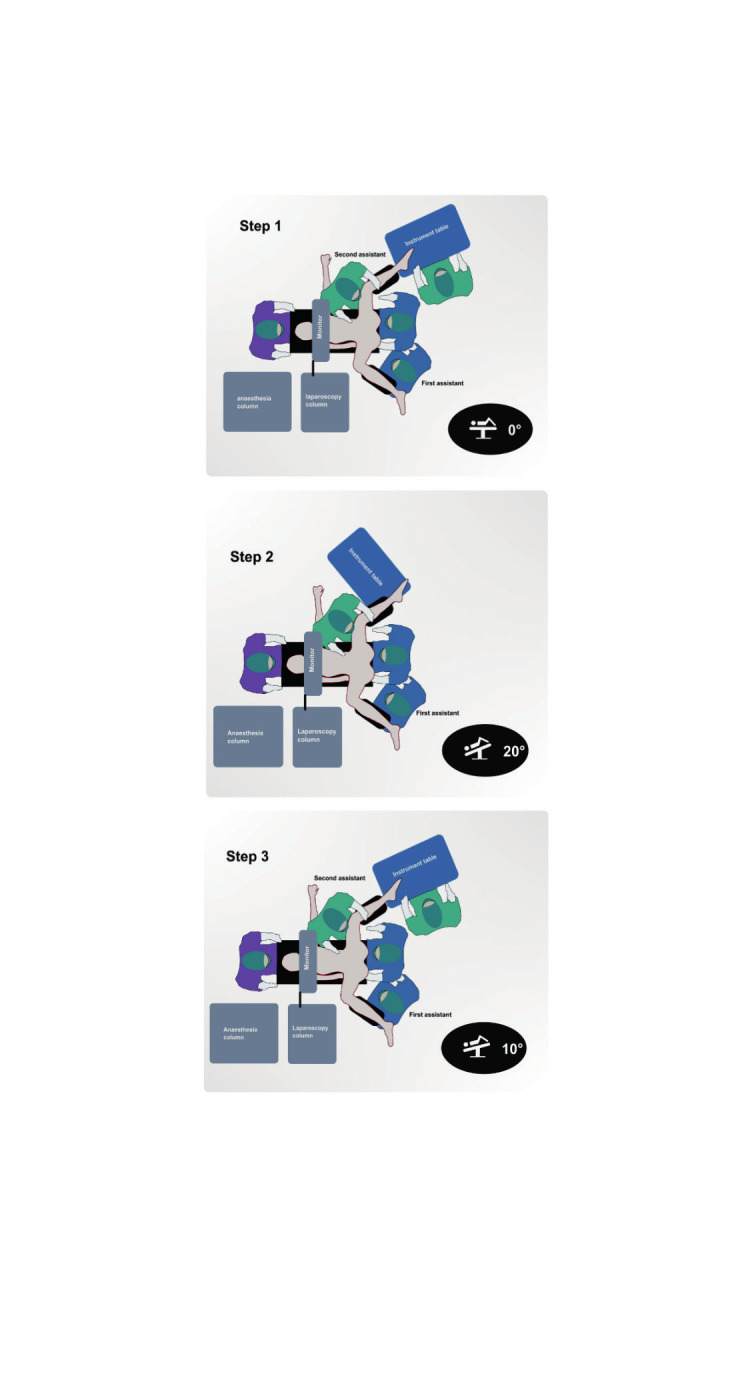
Operative set up during the 3 main steps of the vNOTES hysterectomy: colpotomy and monotrocard placement, laparoscopy, vaginal closure.

A transvaginal device that allows laparoscopic access to the peritoneal cavity is required for vNOTES. The device used was the GelPOINT™ V-Path transvaginal access platform with a diameter of 9.5 cm (Applied Medical, Rancho Santa Margarita, CA, USA) (Figure 1A). The operative technique used (Baekelandt et al., 2019) consists of three operative stages associated with modifications to the installation concerning the degree of Trendelenburg and the placement of the surgical assistants (Figure 2).

The first operative stage is the same as for a conventional vaginal hysterectomy. Access to the peritoneal cavity is achieved through the vaginal route; exposure is achieved with anterior and posterior vaginal retractors and traction on the cervix with a Pozzi forceps. A circumferential colpotomy is performed, followed by opening the vesico-uterine space anteriorly and the cul-de-sac of Douglas posteriorly. The anterior and posterior vaginal retractors are then introduced into the peritoneal cavity, enabling the exposure of the uterosacral ligaments and their suture ligation and cutting at their cervical insertion. The inner ring of the Alexis retractor is then inserted into the peritoneal cavity. The placement of the Alexis retractor is facilitated by sliding it along the vaginal retractor (Figure 1B). Correct anterior and posterior intraperitoneal installation of the device is checked digitally before removal of the vaginal retractors. The Alexis retractor is then put under moderate tension by winding the outer ring two turns. Three trocars are placed on the GelPoint™ platform as laterally as possible at 2, 6, and 10 o’clock before the platform is attached to the proximal end of the Alexis retractor (Figure 1C).

The second operative stage is undertaken into the abdominopelvic cavity (Figure 1D). The CO_2_ gas pressures in the 8–10 mmHg range are used. A standard 10 mm, 0° rigid laparoscope is used through the camera trocar located at 6 o’clock, and standard laparoscopic instruments are inserted through the other two trocars. The laparoscopic instruments used are a Johan-type grasping forceps and a Voyant™ bipolar energy thermofusion instrument (Applied Medical, Rancho Santa Margarita, CA, USA). The path of the ureters is identified by transparency without performing dissection. The surgeon proceeds with the hysterectomy by dissection, section, and coagulation of the different structures in the caudocephalic direction (cervico- vaginal pedicles, uterine pedicles, round ligament, utero-ovarian ligament, lumbo-ovarian ligament or meso salpingo-ovarian ligament depending on the procedure performed on the adnexa). After performing the hysterectomy, the surgeon removes the GelPoint™ device, as well as the uterus, through the vaginal fundal incision.

The third operative stage is closure of the vaginal vault with absorbable thread after exsufflation of the pneumoperitoneum. This is done by suturing the anterior and posterior vaginal slices, including the edge of the peritoneal sheet, after passing two angle stitches through the ends of the uterosacral ligaments that were cut during the first stage of the operation. For the patients who underwent hysterectomy by laparoscopy, the surgery was performed according to a classic technique (Cosson et al., 2016).

### Operative conditions

In the group of patients who underwent operations using vNOTES, the two primary surgeons were two senior surgeons proficient in vaginal and laparoscopic surgery (AA and PC). These surgeons received specific training over two days for vNOTES from an expert surgeon – Jan Baekelandt – in September 2019. They were the primary operators for all the specific times of the vNOTES procedures (GelPoint* placement, laparoscopic time by vaginal approach). When operative circumstances were deemed suitable by the senior surgeon, residents were allowed to perform colpotomy and/or vaginal closure steps under supervision as part of the classical companionship. In the group of patients who underwent operations by laparoscopy, the procedures were performed by one of the senior surgeons of the department. When operative circumstances were deemed suitable by the senior surgeon, residents were allowed to perform any of the operative steps under supervision, as part of the classical companionship.

### Outcome measures

The intra- and postoperative data collected included the type of approach, indication, operative time, uterine weight, intra- and postoperative complications at six weeks, transfusions, pain assessment at day one and length of hospital stay.

The operative time was obtained from operative reports: it was defined as the total duration from skin incision to wound closure. The uterine weight was obtained from pathology reports. Postoperative pain was assessed using the visual analogue scale and obtained from the computerised nursing records; the maximum value recorded on day one was retained. Postoperative complications were described according to the standardised classification of Clavien-Dindo (Katayama et al., 2016; Dindo et al., 2004). Grade I apply to any deviation from the usual procedure that does not require additional drug or surgical management. Grade II indicates complications requiring drug management, including patients who required morphine postoperatively. The following occurrences were also considered Grade II: transfusion, intravenous iron supplementation, urinary catheter placement (in case of acute urine retention) and nasogastric tube placement. Grade III includes all cases for which surgical, endoscopic, or interventional radiology is required. Grade IV refers to life-threatening complications, and Grade V refers to the death of the patient.

### Statistics

Quantitative descriptive values were described by their mean and compared by a Mann–Whitney U test. The qualitative variables were described by their value and as percentages and compared using a chi-square test when the conditions for its application were met and by a Fisher test for the other cases. The results were considered significant when p-values < 0.05 were obtained. The analyses were performed using the SPSS Statistics software version 20.0 (IBM Corp., Armonk, NY 2011, USA).

## Results

Between November 2019 and May 2021, 86 patients who met the selected criteria underwent hysterectomy. Fifty underwent operation using laparoscopy, and 36 using vNOTES. The characteristics of the two groups are summarised and compared in Table I.

**Table I:** Characteristics of women: Comparison between the laparoscopy group and the vNOTES group. Quantitative descriptive values are expressed as mean (minimum-maximum) and qualitative values as number (%).

		Laparoscopy (n=50)	vNOTES (n=36)	p
Age, years		46 (31-74)	47 (32-60)	0.455
Body Mass Index, kg/m^2^		26 (17-37)	27 (18-40)	0.391
No. of vaginal births				
	0	23 (46)	5 (14)	
	1	6 (12)	4 (11)	0.005
	≥2	21 (42)	27 (75)	
Prior caesarean section		18 (36)	4 (11)	0.009
ASA score		1.8	1.8	0.520
Indication for surgery				
Myoma		20 (40)	14 (39)	
Adenomyosis		17 (34)	3 (8,3)	
dysfunctional uterine bleeding		6 (12)	4 (11,1)	-
Essure device removal		5 (10)	12 (33,3)	
Others		2 (4)	3 (8,3)	
Adnexal surgery		50 (100)	34 (94)	0.09
Adnexectomy		12	8	
Salpingectomy		38	26	
Uterine weight, g		281 (72-1695)	238 (48-1450)	0.572

The two groups were similar in terms of age. The age range of all patients was 31–74, with a mean age of 47 years. The BMI range was 16.7–39.8, with a mean BMI of 26.3 kg/m2.

The four most frequent indications for hysterectomy were fibroids (n = 34), adenomyosis (n = 20), menometrorrhagia of other origin (endometrial hyperplasia, polyps) (n = 10), and Essure ® device removal (n = 17). The five remaining indications were hydatidiform mole, symptomatic caesarean scar defect, unexplained pelvic pain, prophylactic surgery for Lynch syndrome, and high-grade cervical dysplasia. The mean uterine weight was 262.2 (SD 275.5) g. Data on uterine weight were missing for four patients in the laparoscopy group.

The intraoperative and postoperative outcomes of each of the two groups are presented and compared in Table II. The mean total operative time was 135.5 (SD 49.6) min. The mean postoperative hospital stay was 1.67 days. There were no cases of transfusion. The mean postoperative pain evaluation on day one was 3.5/10. Intraoperative complications occurred in six patients: Two cases of unplanned oophorectomy were performed for bleeding of the lumbo-ovarian pedicle (one woman in the laparoscopy group and one woman in the vNOTES group). Two cases of emergency laparo-conversion were performed for intraoperative haemorrhage (one woman in the laparoscopy group and one woman in the vNOTES group). Two cases of laparo-conversion occurred in the laparoscopic group because of large poly- fibrous uteri associated with digestive adhesions. Regarding postoperative complications, one patient in the laparoscopy group presented a Grade III complication of re-intervention for pelvic peritonitis following digestive adhesions at the suture of the vaginal vault, with no digestive perforation observed.

**Table II:** Intraoperative and postoperative outcomes. Comparison between the laparoscopy group and in the vNOTES group. Quantitative descriptive values are expressed as mean (standard derivation) and qualitative values as number (%).

		Laparoscopy (n=50)	vNOTES (n=36)	p
Duration of surgery, minutes		149 ± 55	116 ± 34	0.003
Length of hospital stay, days		1.8 ± 1	1.5 ± 0,6	0.207
Visual Analogic Pain score at day 1		3.3 ± 2.2	3.8 ± 2.6	0.458
Transfusion, n		0	0	-
Intraoperative complications, n				
	Total	4 (8)	2 (5.5)	1
	Unplanned ovariectomy	1 (2)	1 (2.7)	1
	Laparoconversion	3 (6)	1 (2.8)	0.636
Postoperative complications, n				
	Total	9 (18)	5 (14)	0.610
	Grade I	3 (6)	2 (5.5)	1
	Grade II	5 (10)	3 (8.3)	1
	Grade III	1 (2)	0	1

## Discussion

This study described the operative outcomes during the implementation of total hysterectomy using vNOTES, which was performed by surgeons experienced in laparoscopy and the vaginal approach in a university hospital. The results presented were favourable and support good feasibility for this technique without additional morbidity compared to laparoscopy.

The operating time was shorter in the vNOTES group. This result may appear surprising at the time of the introduction of a new technique. Possible explanations are (i) the fact that vNOTES cases were easier to operate on, (ii) the more active participation of senior surgeons in the vNOTES procedures, whereas laparoscopic procedures were slowed down by more time dedicated to companionship for residents, and (iii) the vNOTES hysterectomy is by nature a shorter procedure. These results are consistent with Baekelandt et al. (2019) who conducted a randomised trial comparing laparoscopy and vNOTES: this non-inferiority study included 35 patients who underwent successful operations using vNOTES. In that study, all hysterectomies were performed by a single surgeon with experience performing more than 200 procedures using vNOTES. In that randomised trial, postoperative pain and length of hospital stay were reduced in the vNOTES group, and no conversion occurred.

The rates of intra- and postoperative complications observed in our study in the vNOTES group were reassuring, with rates close to those of the laparoscopic group, and the absence of severe life-threatening complications. Although not considered in the Clavien-Dindo classification, the presence of small hematomas on the trocar orifice was mentioned in hospital reports for six of the 50 patients operated on using laparoscopy. The cosmetic advantage of vNOTES compared to laparoscopy due to the absence of scarring is frequently mentioned in the literature, as is the absence of possible complications linked to the trocar ports (haematomas, infections and hernias) (Baekelandt et al., 2019; Lamblin et al.,2021; Su et al., 2012; Lee et al., 2014). The first two studies about vNOTES hysterectomies for benign pathologies were published in 2012 and 2014 (Su et al., 2012; Lee et al., 2014) on a sample of 33 and 137 patients, respectively. These two studies focused on the feasibility and safety of this approach. No conversion was found in the first study, and only seven patients (5.1%) had laparoscopic conversion in the second study. The results of our present study are similar, with only one conversion to laparotomy out of 36 vNOTES procedures (2.7%).

### Large uterus

In the vNOTES group, two uteri weighed more than 500 g, and another uterus weighed more than 1000 g (the latter requiring laparo-conversion for intraoperative haemorrhage). Some publications concluded that vNOTES hysterectomies are feasible for large uteri, but the importance of a longer learning curve to operate on these more complex cases was underlined. Lee et al. (2014) published a case series, in which 34% of patients included had a uterus weighting over 500 g and 5.4% over 1000 g. A more recent study published by Wang et al. (2020) focused on 39 vNOTES hysterectomies of uteri over 1 kg. The average uterine weight was 1141 g (maximum rank 1700 g), with 90% operative success using vNOTES. In these two studies, the advantage of dividing uterine vessels at the beginning of the operation was underlined. This early haemostasis, made possible by a caudo- cephalic uterine approach, could be a factor limiting blood loss in the management of fibroid uteri by vNOTES.

### Surgical training

The vNOTES technique combines two surgical techniques already mastered by most gynaecological surgeons: the vaginal route and laparoscopy. However, the technique has specificities that require prior training, including operative setting, placement of surgical assistants, vaginal port placement and ascending laparoscopic vision. The two primary surgeons in the present study performed their first vNOTES hysterectomies within a month of the two- day training received in 2019. Both surgeons were within in their initial learning curve for the specific vNOTES hysterectomy procedure. Because some of the common vaginal gestures (i.e., colpotomy and vaginal closure) were performed by supervised residents, reliable data for the learning curve of the two primary senior surgeons cannot be provided. The learning curve of a single surgeon was studied in two recent publications. Lauterbach et al. (2020) showed an improvement in operative time and a decrease in blood loss between the first 10 procedures and the next. Wang et al. (2019) studied the learning curve of 240 vNOTES hysterectomies by one surgeon on the sole criterion of operative time. This study suggested that it is necessary to perform 20 vNOTES hysterectomies to acquire basic skills, and about 80 procedures to fully master the surgical technique allowing for the operation of more complex cases. The learning curve will need to be further investigated in future studies with multiple operators.

### Limitations and advantages of the vNOTES technique

The main technical specifics of vNOTES are the approach to the peritoneal cavity and the placement of the GelPOINT™ device. Certain clinical situations are likely to lead to complications during these steps of the procedure. An expert consensus has identified the risk of intra-pelvic adhesions and anatomical distortion (endometriosis, pelvic radiotherapy and inflammatory bowel diseases) as the main contraindications to vNOTES (Kapurubandara et al., 2021). On the other hand, a history of one or more caesarean sections was not a contraindication to vNOTES, according to these experts.

The use of laparoscopic tools confers several advantages on vNOTES compared to the traditional vaginal route: a better field of vision for the whole surgical team, good accessibility to the pelvic cavity due to the long laparoscopic instruments and improved ergonomics for the surgical assistants. These advantages could facilitate concomitant management of the adnexa during vNOTES hysterectomy and broaden the indications of this approach. Of the 36 vNOTES hysterectomies performed in this study, 94% had additional salpingectomy or oophorectomy, whether for a specific indication or as part of a prophylactic indication (Touboul et al., 2021). Moreover, satisfactory control of the haemostasis of the infundibulopelvic vascular pedicles was also facilitated at the end of the operation, thanks to the laparoscopic view.

### Limitations

The limitations of this study are inherent in its retrospective nature. Although the two group’s uterine weights were similar, the characteristics of the operated patients could be considered more favourable in the vNOTES group. Thus, more patients in the vNOTES group had a history of vaginal deliveries, and fewer had a scarred uterus. Additionally, one-third of the indications in the vNOTES group concerned Essure ® removal involving a small uterine volume with a low risk of adhesions. Given the difference in patient characteristics between the vNOTES group and the laparoscopy group, statistical comparison should be interpreted with caution. However, presenting the outcomes of patients managed by laparoscopy during the same period provides the reader with perspective on the way our team carried out this classic minimally invasive procedure.

## Conclusion

During the introductory period of the vNOTES hysterectomy technique in a teaching hospital, a low rate of complications was observed. Operative outcomes were similar to those of patients who underwent conventional laparoscopic hysterectomy. Two experienced surgeons were the primaries on the vNOTES procedures, with the active participation of residents as part of their classical companionship. These outcomes are reassuring regarding patient safety while introducing vNOTES in an academic hospital. Further studies are needed to explore the learning curve of trainees for specific steps of this technique.
